# Cellular hierarchy framework based on single-cell and bulk RNA sequencing reveals fatty acid metabolic biomarker MYDGF as a therapeutic target for ccRCC

**DOI:** 10.3389/fimmu.2025.1615601

**Published:** 2025-06-05

**Authors:** Ning Wang, Ziyu Xu, Lina Zhang, Yanfang Lu, Yanliang Wang, Lei Yan, Huixia Cao, Limeng Wang, Fengmin Shao

**Affiliations:** ^1^ Department of Nephrology, Zhengzhou University People’s Hospital, Henan Provincial People’s Hospital, Zhengzhou, China; ^2^ Henan Provincial Key Laboratory of Kidney Disease and Immunology, Henan Provincial Clinical Research Center for Kidney Disease, Henan Provincial People’s Hospital, Zhengzhou, China

**Keywords:** fatty acid metabolism, ccRCC, MYDGF, ScRNA-seq, machine learning

## Abstract

**Background:**

Fatty acid metabolism (FAM) reprogramming is a prominent feature of clear cell renal cell carcinoma (ccRCC). Nevertheless, the effect of FAM reprogramming on the heterogeneity and prognosis of ccRCC individuals remains insufficiently understood.

**Methods:**

We utilized single-cell sequencing and spatial transcriptomics to investigate the heterogeneity of FAM in ccRCC comprehensively. Functional enrichment algorithms, including AUCell, UCell, singscore, ssGSEA, and AddModuleScore, along with hdWGCNA analysis, were used to identify hub genes influencing high FAM of ccRCC. Machine learning methods were then applied to pinpoint the optimal feature gene. The function of the selected genes in FAM was validated through clinical samples and cellular functional experiments.

**Results:**

The results revealed significant upregulation of FAM in malignant epithelial cells. Through five distinct enrichment scoring methods and hdWGCNA analysis, we redefined a gene set related to increased FAM at the single-cell level. By the integration of this gene set with bulk transcriptomic data and the application of machine-learning algorithms, we found four candidate genes—MYDGF, ZNHIT1, HMGN3, and ARL6IP4—that were linked to ccRCC progression. Bulk RNA sequencing validated their increased expression in ccRCC individuals, underscoring their diagnostic and prognostic potential. Single-cell analysis further revealed that these genes were primarily upregulated in malignant epithelial cells, emphasizing their cell-specific roles in ccRCC. It was verified that MYDGF could promote cell proliferation, migration and invasion while inhibiting cell apoptosis. Functional experiments further confirmed that MYDGF is a key FAM-related biomarker that enhances lipid deposition by suppressing fatty acid oxidation, thereby accelerating tumor progression.

**Conclusions:**

MYDGF was identified as a FAM-related oncogenic biomarker that promotes ccRCC progression by inhibiting fatty acid oxidation. Our findings elucidated the cellular hierarchy of ccRCC from the perspective of FAM reprogramming and may offer new insights and therapeutic targets for future ccRCC treatments.

## Introduction

Renal cancer ranks as one of the most prevalent malignancies in the urinary system, with 155,702 global deaths reported in 2022. The occurrence of kidney cancer has also been gradually elevating, with 434,419 new cases worldwide in the same year (1–3). Clear cell renal cell carcinoma (ccRCC) is the most prevalent and aggressive subtype, resulting in almost 60% to 80% of all primary cases (4). The effectiveness of current clinical treatments for ccRCC, including surgery, conventional chemotherapy, targeted therapy, and immunotherapy, is constrained by both inter- and intratumor heterogeneity (5). Over one-third of ccRCC individuals experience relapse and metastasis following surgery, with a poor prognosis for metastatic cases, reflected by a five-year survival rate of just 10% (6). Thus, investigating the cellular mechanisms driving ccRCC progression and detecting innovative therapeutic targets is essential for enhancing the outcomes of patients.

Metabolic reprogramming is a defining feature of ccRCC, marked by the aberrant lipid droplet accumulation within tumor cells (7). Lipid storage is a crucial adaptive mechanism in tumors rather than a mere bystander effect in tumor growth (8). Recent investigations have shown that lipid storage is linked to abnormal fatty acid metabolism (FAM) (9, 10). *De novo* fatty acid synthesis, uptake, and inhibition of fatty acid oxidation (FAO) contributed to lipid storage (11). Storing excess fatty acids is important for maintaining endoplasmic reticulum function and preventing lipotoxicity by reducing harmful reactive oxygen species from lipids (12, 13). Moreover, enhanced lipid storage may confer additional advantages to ccRCC, as elevated phosphatidylcholine levels support cell membrane fluidity, thereby promoting metastatic potential (14). These outcomes underscore the role of lipid storage in ccRCC progression and emphasize the need to investigate the molecular mechanisms behind altered FAM further.

Despite the growing recognition of the critical function of FAM in the ccRCC pathogenesis and progression, the determination and validation of hub regulatory genes are still challenging. Conventional experimental techniques often fall short in detecting cell-specific gene expression and metabolic processes, which are essential for comprehending the heterogeneous regulation of FAM in ccRCC. Single-cell RNA sequencing (scRNA-seq) provides insight into tumor cell heterogeneity at single-cell resolution, enabling the detection of rare cell populations, characterization of cellular subtypes, lineage tracing, and the discovery of novel biomarkers (15). This technology offers new perspectives on tumor metabolic reprogramming. Additionally, spatial transcriptomics (ST) complements single-cell omics by characterizing cellular components in spatial environments, providing high-throughput strategies to examine tumor heterogeneity (16). However, the limited sample size in scRNA-seq datasets restricts the comprehensive exploration of the relationship between metabolic reprogramming at the cellular level and interpatient heterogeneity, factors that may contribute to poor prognoses in ccRCC. Therefore, integrating multidimensional data for joint analyses is essential to compensate for unreliable or missing information from single-omics data, facilitating the discovery of novel disease indicators and more accurate therapeutic targets.

Herein, scRNA-seq, ST, and bulk RNA-seq data were utilized to examine the FAM function in ccRCC comprehensively. FAM heterogeneity was initially identified at the single-cell level, revealing remarkable variability across cell types, with a notable increase in malignant tumor cells. By applying various machine learning algorithms to bulk RNA-seq data, we identified key genes associated with FAM upregulation and examined their involvement in ccRCC pathology and progression. Finally, the regulatory role of MYDGF in FAM was validated through *in vitro* experiments. Our outcomes offer important visions into the genetic underpinnings of FAM and provide a foundation for future studies and potential therapeutic approaches for ccRCC.

## Materials and methods

### Data collection

The scRNA-seq, such as 19 ccRCC samples, were acquired from the GEO database using accession ID: GSE207493 (17). The bulk RNA-seq data and the clinical characteristics of ccRCC were accessed from TCGA databases. One spatial transcriptomics RNA sequencing (stRNA-seq) sample of ccRCC with the accession ID GSE175540 was involved in this investigation. Detailed information about all the datasets used in this study was provided in **Supplementary Table S1**. A total of 323 FAM-associated genes were brought from the KEGG, REACTOME, Hallmark MSigDB v5.2, and earlier investigations (18) (**Supplementary Table S2**).

### Data analysis

For the scRNA-seq data processing, high-quality cells were retained, defined as those with fewer than 20% mitochondrial gene expression and the expression of greater than 200 genes. We concentrated on genes with expression levels between 200 and 7,000, detected in a minimum of three cells. A total of 154,130 suitable cells were included for additional analysis. Subsequently, the Seurat pipeline was employed for integrating data (19). The remaining cells were normalized and scaled using a linear regression model using the “Log Normalization” approach. The top 3,000 extremely variable genes were determined via the “FindVariableFeatures” function. Data were then reduced using principal component analysis (PCA). Correcting the batch effects between samples was conducted via the “Harmony” package (20) to ensure that they did not interfere with downstream analyses. Cell clustering was carried out via the “FindClusters” function. The annotation of cell clusters involved the identification of highly expressed and uniquely expressed genes and known canonical cellular markers.

### Infer the malignant epithelial cells

Using the inferCNV R program, copy number variation (CNV) profiles were created to identify cancer cells with clonal, substantial chromosomal CNV (21). The CNV score was measured as the mean of the squared CNV values for all chromosomes. Malignancy or non-malignancy labels were allocated based on distributing the malignancy scores in relation to the reference and the identification of bimodal features.

### Gene set scoring algorithm in scRNA-seq

Five distinct algorithms were employed to assess FAM activity in scRNA-seq datasets: AUCell, UCell, singscore, ssGSEA, and AddModuleScore (22, 23). Based on the quartile of the resulting FAM activity scores, malignant cells were categorized into three categories: low FAM activity state (LFS), dynamic transition FAM activity state (DTFS), and high FAM activity state (HFS). The “FindMarkers” function was then employed to detect differentially expressed genes (DEGs) involved in upregulating FAM.

### CytoTRACE analysis

We conducted CytoTRACE analysis using default parameters, a method that forecasts differentiation states using scRNA-seq data, predicated on the assumption that transcriptional diversity diminishes during differentiating (24). This analysis was employed to quantify the progressive status of every cell by examining alterations in gene expression. Upon completion of the CytoTRACE algorithm, each cell was given a score reflecting its stemness within the dataset. The CytoTRACE R package was used to calculate scores for malignant cells, with values of 0-1. Greater scores signified greater stemness (low differentiation) than lower scores, indicating reduced stemness.

### Cell communication

The data analysis of gene expression and the exploration of potential variations in cell-cell communication networks were conducted using CellChat (25). Subsequent to the conventional CellChat pipeline, we utilized the default CellChatDB to identify ligand-receptor interactions. By examining the overexpression of ligands and receptors within distinct cell populations, we were able to infer interactions that are specific to individual cell types. Moreover, we observed an increase in ligand-receptor interactions that correlated with the overexpression of certain ligands or receptors.

### Gene set variation analysis

The GSVA R program was employed to conduct GSVA in order to investigate potential biological pathways that differ across various groups (26), with marker pathways sourced MSigDB database. GSVA was applied to each cell type to estimate pathway activity, and the average gene expression for all subtypes was computed. The differences in activity scores were subsequently utilized for the quantification of the variation in pathway activity across various cell subtypes.

### hdWGCNA

The hdWGCNA method facilitates the analysis of weighted gene co-expression networks in high-dimensional datasets, including scRNA-seq data, permitting the exploration of gene co-expression levels and network dynamics within cell populations (27–29). Utilizing hdWGCNA R package (30), scale-free networks were constructed at the single-cell level. A threshold of > 0.85 was set for the scale-free topology model fit, with a soft threshold of 12 chosen to optimize connectivity within the network. A co-expression network was created via the “ConstructNetwork” function, and the “UCell” method was employed to detect the most relevant modules for HFS cells. The outcomes of the shared candidate genes were recognized through hdWGCNA analysis, and DEGs performed further assessment.

### Functional enrichment analysis

To examine the roles and mechanisms of candidate genes, gene ontology (GO) and disease ontology (DO) enrichment analyses were carried out utilizing the clusterProfiler R package (31).

### Screening of optimal feature genes

An in-depth analysis of the previously screened candidate genes was conducted to detect optimal feature genes (OFGs) associated with FAM activity. To select the most relevant genes, we first conducted univariate Cox regression analysis in the TCGA-KIRC cohort to detect genes significantly correlated with ccRCC overall survival (*P* < 0.05). To guarantee this selection reliability, we employed a bootstrap methodology by sampling 80% of the individuals 1000 times and retained only those genes with *P* < 0.05 occurring more than 800 times. Three machine-learning algorithms were subsequently applied to detect the most accurate feature genes: the Boruta algorithm, the Random Forest (RF) survival algorithm, and the XGBoost algorithm (32–35). These algorithms were chosen for their complementary intensities in selecting features, optimizing models, and mitigating biases from relying on a single algorithm, collectively enhancing the strength and precision of detecting ccRCC-specific genes. Finally, the genes common to all three algorithms were chosen as hub FAM-related genes in ccRCC and represented by using a Venn diagram.

### Validation of the OFGs

We conducted validation at both the scRNA-seq and bulk RNA-seq levels to evaluate the precision of our results. We investigated the enrichment of the OFGs across different cell types by examining annotated scRNA-seq data. This research revealed the particular cell types in which the OFGs contributed to the upregulation of FAM activity. The influence of characteristic genes on overall survival was evaluated via Kaplan-Meier (KM) survival analysis. The expression of the OFGs in TCGA-KIRC samples was validated via the Wilcoxon rank sum test. The receiver operating characteristic (ROC) and area under the ROC (AUC) curves were utilized to verify the predictive value of these feature genes.

### Cell culture and human tissues

Human renal cell line HK-2 was obtained from the American Type Culture Collection (Manassas, USA) and cultivated in DMEM/F12 medium (Gibco, Brazil) supplemented with 10% fetal bovine serum (FBS, Biological Industries, Israel). All human ccRCC cell lines (ACHN, A498, CAKI, 769-P, and 786-O) were acquired from the National Collection of Authenticated Cell Cultures, Chinese Academy of Sciences (Shanghai, China). The 786-O and 769-P cell lines were cultured in RPMI-1640 medium (Invitrogen, USA) with 10% FBS and 1% penicillin-streptomycin (Solarbio, China). CAKI cells were cultivated in McCoy’s 5A medium (Biological Industries, Israel) with 10% FBS and 1% penicillin-streptomycin, while ACHN and A498 cells were maintained in MEM medium (iCell Bioscience, China) with the same supplements. All cells were kept in an incubator with 5% CO_2_ at 37°C and humidified conditions.

Cancerous and matched normal kidney tissues (minimum 3 cm away) were gathered from individuals undergoing radical nephrectomy at Henan Provincial People’s Hospital (Zhengzhou, China). All individuals diagnosed with ccRCC did not undergo radiotherapy, chemotherapy, or immunotherapy prior to surgery. The investigation received approval from the Human Research Ethics Committee of Henan Provincial People’s Hospital (Approval No. 2019-44), and written informed consent was obtained from all participants.

### Western blot

The trials were conducted as earlier described (36). The primary antibodies used in the western blot (WB) experiments were detailed in **Supplementary Table S3**. Horseradish peroxidase (HRP)-linked secondary antibody (A0208 at 1/2000 dilution) was acquired from Beyotime Technology (Shanghai, China).

### Quantitative real−time PCR and RNA isolation

The trials were carried out as earlier described (37). The following are the primer sequences. MYDGF forward: 5’-GGCGTCGTGCATTCCTTCT-3’, Reverse:5’-CCATTGCTCATTGGTCCCTC-3’; β-actin Forward: 5’- GAGAAAATCTGGCACCACACC-3’, Reverse: 5’- GGATAGCACAGCCTGGATAGCAA-3’.

### Immunohistochemistry staining

After fixing renal tissues in 4% paraformaldehyde, dehydration, and paraffin-embedding were conducted. The tissue blocks were sectioned into 5 μm slices, then deparaffinization with xylene and rehydration through a graded ethanol series were conducted. Boiling sections in sodium citrate buffer for 15 min was performed for antigen retrieval, and then cooling to room temperature was conducted. A 3% H_2_O_2_ was used to block endogenous peroxidase activity for 10 min, and then 5% BSA was used to block sections for 30 min. The MYDGF antibodies (Proteintech, 11353-1-AP at 1/500 dilution) were maintained overnight at 4°C. Next, adding secondary antibodies was conducted for 1 h at room temperature. Detection and quantification were carried out via DAB staining, and the nuclei were counterstained with hematoxylin.

### Small interference RNA transfection

The small interference RNAs (siRNAs) were acquired from Sangon Biotech Co., Ltd (Shanghai, China) and transfected using Lipofectamine™ RNAiMAX Transfection Reagent (Thermo Fisher Scientific, Cat#13778030) as per the manufacturer’s guidelines. In brief, the cells were cultured to 50–60% confluence and transfected with negative control (NC) and knockdown (siMYDGF). 5 μL siRNA mix and 5 μL RNAiMAX were added to 125 μL Opti-MEM and incubated at 25°C for 10 min. The resulting mixture was then gently added to the medium to prepare siRNAs at approximately 50 nM. After 48 hours of transfection, the cells were harvested for subsequent analysis. The sequences were as follows: MYDGF siRNA-1: AUACGUGUAUGUUCACUUA; MYDGF siRNA-2: UUCAAAUGCGGCUUUAGAG.

### Cell counting kit-8 assay

At 48 h after siRNA oligo transfection, cells were plated into 96-well plates at 2,000 cells per well. Cell viability was evaluated at 0, 24, 48, 72, and 96 h via the Cell counting kit-8 (CCK-8) assay (Dojindo, Kumamoto, Japan) as per the manufacturer’s guidelines. In brief, CCK-8 reagent was applied, and a 2-h incubation of cells was conducted at 37°C before measuring absorbance at 450 nm.

### Wound healing and transwell assay

Cells that were grown to confluency with or without MDGF suppression were used for the wound healing assay. A 200 µL pipette tip was used to produce the wound. After 48 h at 37°C, photographs were taken at 0 and 48 h. For the Transwell assay, 5 × 10^4^ cells with or without MYDGF suppression were cultured in the upper chambers of 24-well Transwell inserts (8 µm; Corning, USA) with or without matrigel for the invasion assay or migration assay in a serum-free medium. The bottom chambers contained medium with 10% FBS. Matrigel was used for the invasion assay, while the migration assay was conducted without Matrigel. Subsequent to 48 h, fixing cells was conducted with 4% paraformaldehyde, followed by staining with 1% crystal violet and imaging via a Macro Zoom Fluorescence Microscope (Olympus, MVX10).

### Apoptosis analysis

Flow cytometry was utilized to detect apoptotic cells using an Annexin V-FITC Apoptosis Detection Kit (Elabscience, China). Briefly, resuspending 5 × 10^5^ cells was conducted in 500 μL of Annexin V-FITC binding buffer, followed by adding 5 μL of Annexin V-FITC and 5 μL of propidium iodide (PI). After a 15-min incubation at 25°C, apoptosis was assessed by flow cytometry (FACSAria III, BD, USA).

### Nile red staining

In accordance with the manufacturer’s protocol, lipid droplets in cells were detected using a lipid fluorescence staining kit (Nile Red method, Solarbio, China) after MYDGF knockdown. First, the cells were washed with PBS and fixed with 4% paraformaldehyde. A staining solution (500 μL) was then applied, and the cells were incubated in the dark for 15 minutes. Fluorescence images were captured using an EVOS FL microscope (Thermo Fisher Scientific, USA).

### Statistical analysis

Data analysis and graphing were performed via R version 4.3.1 and GraphPad Prism 9.0. The Wilcoxon test or t-test was utilized to compare between two groups, and one-way ANOVA for several groups. Pearson’s correlation coefficient assessed variable correlations. Statistical significance was denoted by *P* values < 0.05.

## Results

### Profiling the heterogeneity of FAM in ccRCC

**Figure 1:**
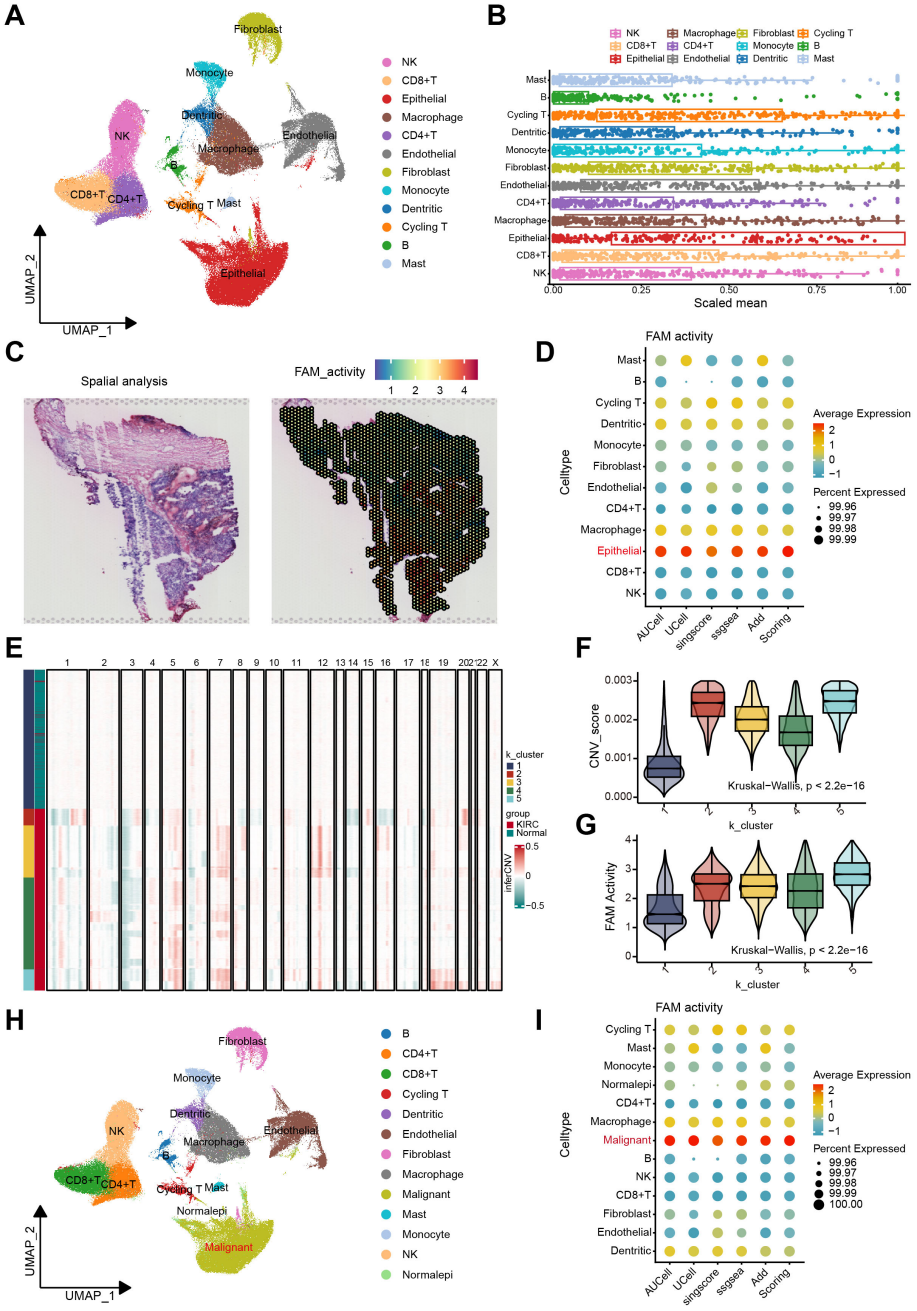
The FAM activity was elevated in malignant epithelial cells of ccRCC. **(A)** The UMAP plot of the ccRCC cells, colored by cell type in GSE207493. **(B)** Boxplots illustrated the scaled mean expression of FAM signatures across various cell types. **(C)** H&E staining and heatmaps of the spatial distribution of FAM activity from the GSE175540. **(D)** The Bubble plot showed enrichment scores of FAM gene sets for all cell types via AUCell, UCell, singscore, ssGSEA, AddModulescore, and Scoring score. **(E)** K-means clustering of inferred CNVs to acquire malignant cells. **(F)** Difference of CNV score for 5 clusters. **(G)** Comparison of FAM activity across 5 clusters. **(H)** Cell reannotation further identified malignant cells within the ccRCC. **(I)** The Bubble plot revealed that malignant cells exhibit higher FAM enrichment scores, as determined by multiple algorithms.

Before proceeding with additional assessment, quality control was conducted on all comprised samples (**Supplementary Figure S1A**). Every sample underwent batch effect correction (**Supplementary Figure S1B**), and the overall distribution was stable, confirming the suitability of the data for downstream analysis. Using the Seurat pipeline, all cells were classified into 29 subgroups (**Supplementary Figure S1C**). Classical marker genes were used for cell annotation, resulting in the identification of 12 cell types, including monocytes, macrophages, fibroblasts, CD8+ T, CD4+ T, cycling T, NK, B, dendritic, mast, endothelial, and epithelial cells (**Figure 1A**). The accuracy of the cell type annotations was validated through a bubble plot of marker genes (**Supplementary Figure S1D**).

We assessed the activity of FAM across 12 cell types using the acquired gene set. The boxplot analysis revealed that FAM activity showed a significant increase in epithelial cells compared to other cell types (**Figure 1B**). Additionally, we examined the spatial distribution of FAM activity using stRNA-seq data. Notably, regions with high FAM activity were predominantly located in the central core of ccRCC tumor (**Figure 1C**). These findings suggest considerable heterogeneity in FAM across different cell types in ccRCC. To further explore this heterogeneity, we quantified FAM activity in every cell via the AUCell, Ucell, singscore, ssgsea, and AddModuleScore algorithms (**Supplementary Figures S2A–C**). All algorithms confirmed that FAM activity was highest in epithelial cells (**Figure 1D**). We then employed inferCNV to identify malignant cells among epithelial cells. The CNV profiling results revealed heterogeneity between epithelial and reference cells (**Supplementary Figure S2D**). Using unsupervised K-means clustering with five clusters, we distinguished cells with increased and decreased CNV (**Figure 1E**). Cluster 1 exhibited the lowest CNV score, containing a higher proportion of normal tissue-derived epithelial cells. Therefore, healthy epithelial cells were identified in cluster 1, and malignant cells in the other clusters (**Figure 1F**). Notably, FAM activity was significantly elevated in clusters 2 through 5, indicating a marked increase in malignant cells (**Figure 1G**). The UMAP plot illustrated the distribution of normal and malignant epithelial cells (**Figure 1H**). After identifying the malignant cells, we reassessed FAM activity using the same method (**Supplementary Figure S2E**). The results demonstrated that FAM activity was significantly higher in malignant cells (**Figure 1I**).

### Unraveling the complexities of FAM in malignant cells

**Figure 2:**
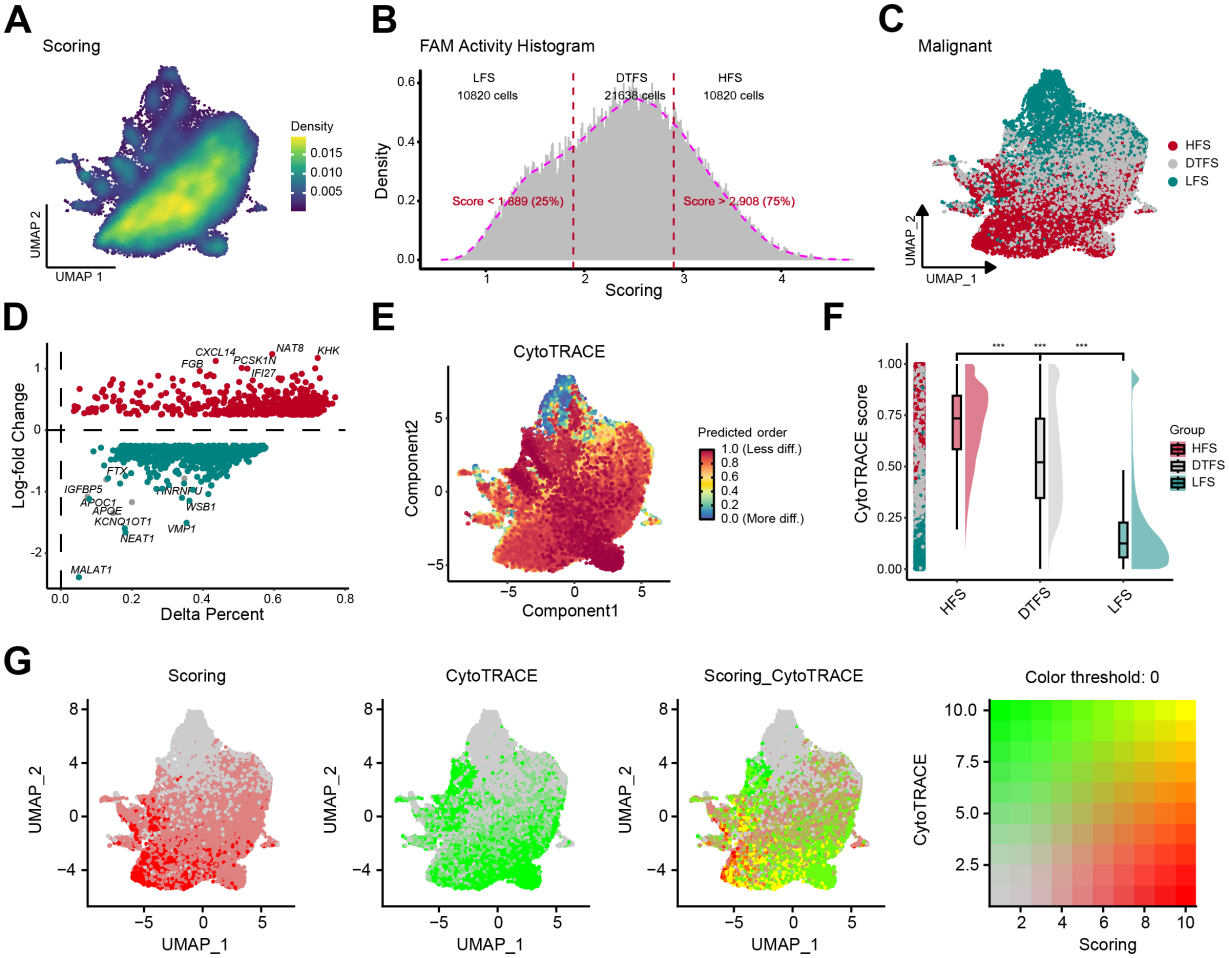
Determination and characterization of FAM-related malignant cells. **(A)** The UMAP plot revealed the heterogeneity of FAM activity in malignant cells. **(B-C)** Based on FAM activity scores, malignant cells are categorized into three groups: LFS, DTFS, and HFS. **(D)** The percentage difference (representing the proportion of cells) and log-fold change were calculated using the Wilcoxon rank-sum test for DEGs between the HFS and LFS groups. **(E)** The UMAP plot displayed dedifferentiation scores inferred from CytoTRACE. **(F)** A raincloud plot of CytoTRACE scores for HFS, DTFS, and LFS malignant cells. The center of the box plot represents the median values, and the box bounds correspond to the 25th and 75th percentiles. **(G)** The correlation between the CytoTRACE score and FAM score.

The UMAP plot illustrated the distribution of FAM activity in malignant cells (**Figure 2A**). We observed that FAM activity showed heterogeneity even within malignant epithelial cells. Therefore, we categorized malignant cells into three groups according to the quartiles of their FAM activity scores: LFS, DTFS, and HFS (**Figures 2B, C**). We then conducted DEG on the upregulated genes of FAM between HFS and LFS groups (**Figure 2D**). The DEG outcomes detected 606 genes that participated in upregulating the FAM activity (**Supplementary Table S4**). CytoTRACE analysis illustrated a significant elevation in tumor stemness features in the HFS group compared to LFS (**Figures 2E, F**), with a strong positive correlation between FAM activity scores and CytoTRACE scores (**Figure 2G**, **Supplementary Figure S3**).

### Function analysis of FAM in scRNA-seq data

**Figure 3:**
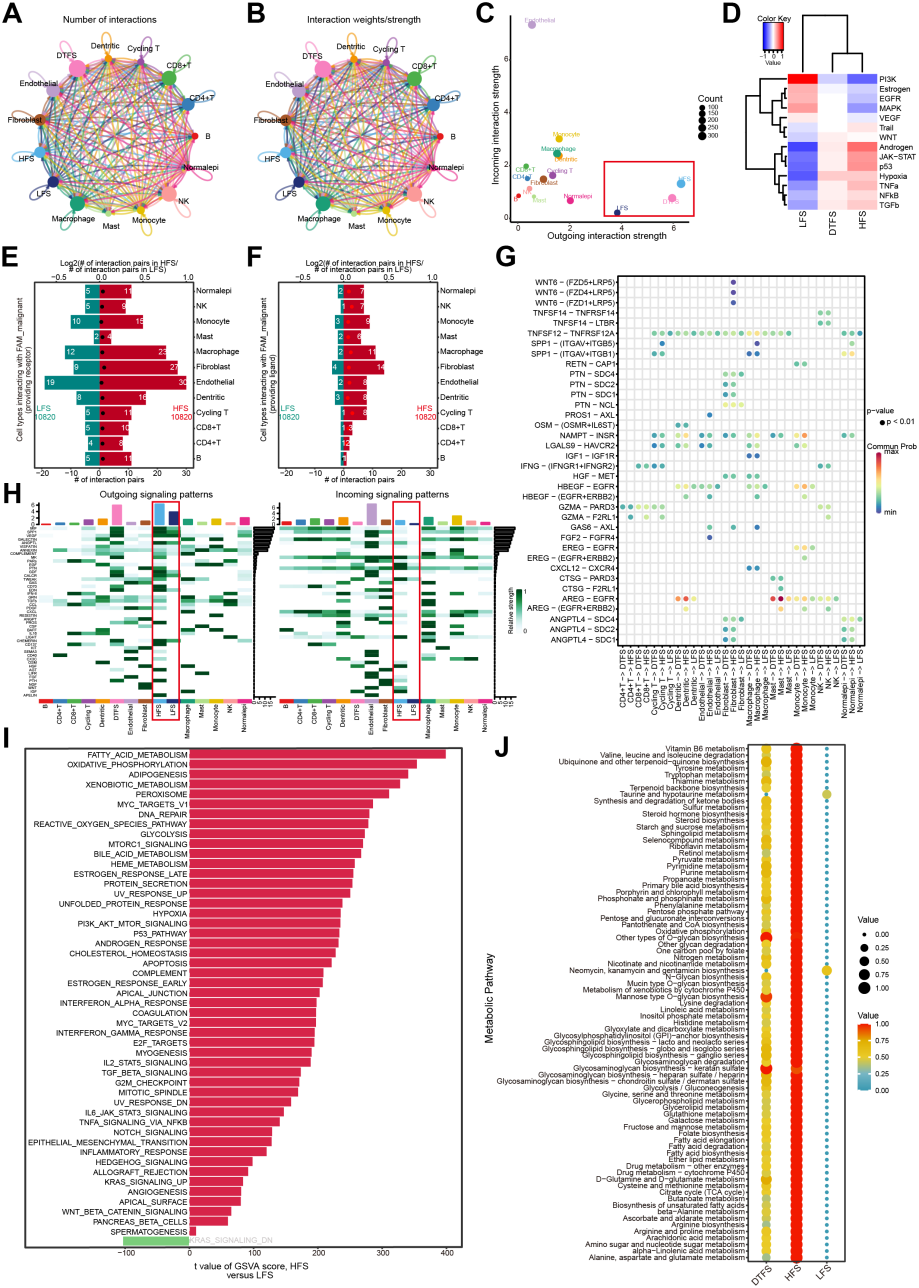
Function analysis of HFS, DTFS, and LFS malignant cells according to scRNA-seq data. **(A, B)** Cellchat analysis of malignant cells from the HFS, DTFS, and LFS groups, along with other cell types. Both interaction counts and strengths are presented. **(C)** Interaction dynamics across different cell types. **(D)** A heatmap illustrated the differences in signaling pathways among the three groups. **(E-F)** Bar plots showed the number of interactions between HFS/LFS malignant cells and other cell types. **(G)** Cellular communication between HFS, DTFS, LFS malignant cells, and other cell types. **(H)** Heatmap of the cell-cell communication network for both incoming and outgoing signaling analysis. **(I)** Variations in hallmark gene set pathway activities, scored per cell by GSVA. **(J)** Differences in metabolism-related pathways among LFS, DTFS, and HFS malignant cells.

We conducted an extensive assessment of the interactions between LFS, DTFS, HFS, and other cell types using CellChat integrated with scRNA-seq data. The cell communication results reflected both the count and intensity of interactions between LFS, DTFS, and HFS cells and other cell types (**Figures 3A, B**). Our analysis revealed that HFS cells are more efficient in signal transmission. The overall level of intercellular communication demonstrated dynamic growth across the three groups (**Figure 3C**). For additional evaluation of the communication differences between HFS and LFS cells, the expression of receptors and ligands was assessed. The results showed that, compared to LFS cells, HFS cells showed a significant elevation in the count of potential ligand-receptor pairs with other cells (**Figures 3E–G**). Heatmap analysis indicated a higher probability of communication in HFS cells (**Figure 3H**).

Additionally, analysis of hallmark pathways revealed greater variation between HFS and LFS cells. A direct comparison between HFS and LFS cells identified FAM as the most enriched signature in HFS cells (**Figure 3I**). Several signaling pathways related to metabolism and tumorigenesis, such as glycolysis, xenobiotic metabolism, and the P53 signaling pathway, were also activated in HFS cells (**Figures 3D, I**). Metabolic pathway analysis revealed an overall increase in metabolic activity in HFS cells (**Figure 3J**). These findings suggested that HFS cells possess enhanced cell communication capabilities and activate more tumor- and metabolism-related pathways.

### Identification of critical modules associated with HFS malignant cells through hdWGCNA

**Figure 4:**
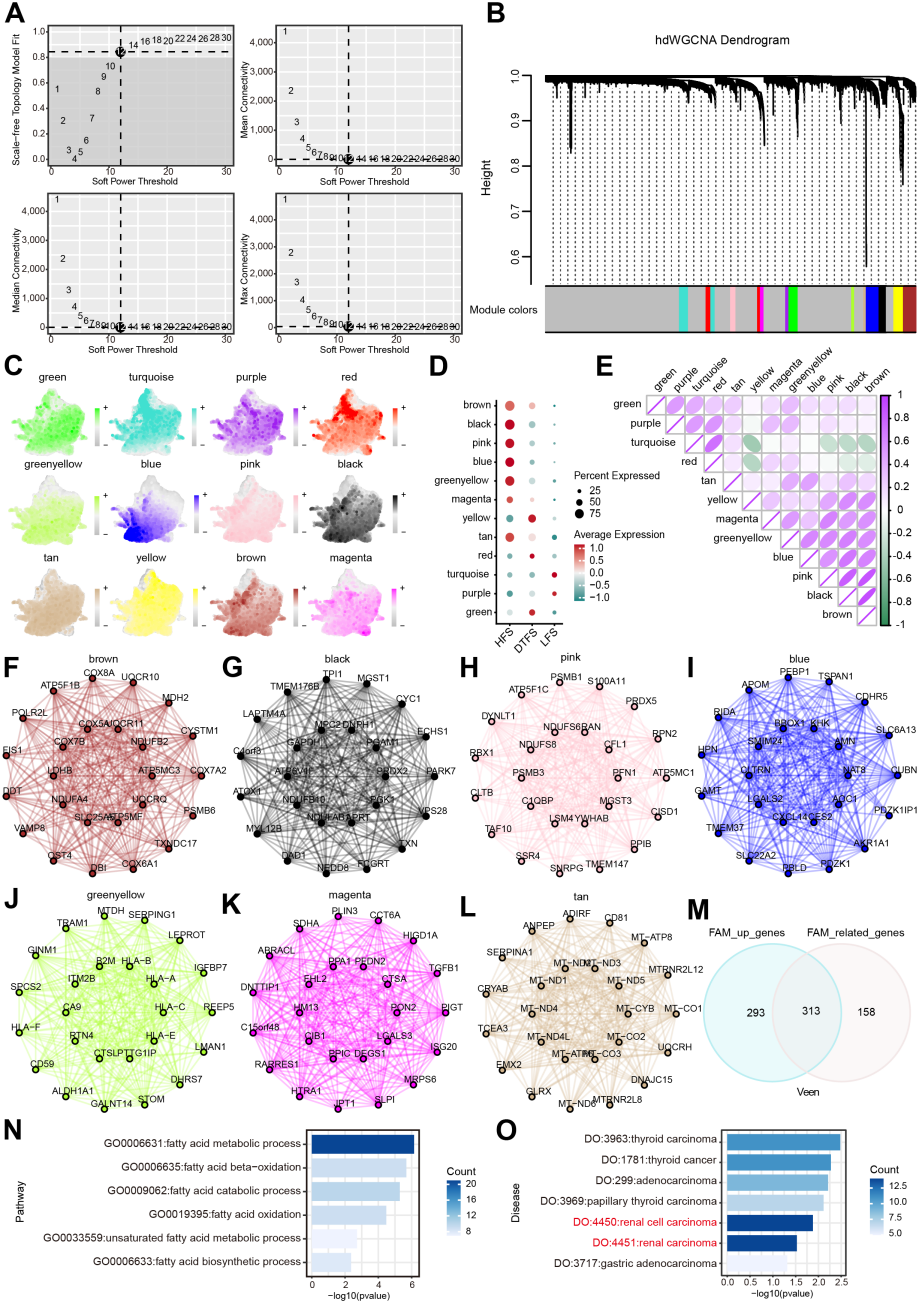
Determination of the essential modules linked to HFS malignant cells by hdWGCNA. **(A)** Scale-free fit index and mean connectivity plot for numerous soft threshold powers. **(B)** Hierarchical cluster tree of gene modules determined through WGCNA. Twelve modules were determined as presented in the hdWGCNA dendrogram. **(C)** UMAP plots depicting feature scores for each module in hdWGCNA. **(D)** The bubble plot exhibited the scores acquired by 12 modules. **(E)** Correlation analysis between different models. **(F–L)** The first 25 eigengenes of the selected module. **(M)** The outcomes of the Venn diagram of hdWGCNA analysis and DEG analysis. **(N)** GO enrichment analysis of the overlapping genes. **(O)** DO enrichment analysis of the overlapping genes.

The hdWGCNA pipeline was employed to identify co-expressed gene modules in HFS malignant cells. Using an optimal soft threshold of 12, a scale-free co-expression network was created, leading to the detection of 12 distinct modules (**Figures 4A, B**). The UMAP plot provided an intuitive and comprehensive visualization of the distribution of cell subsets in every module (**Figure 4C**). The bubble plot revealed significant correlations between the black, blue, brown, green-yellow, magenta, pink and tan modules and HFS malignant cells (**Figure 4D**). The heatmap visualized the correlation between different modules (**Figure 4E**). **Figures 4F–L** illustrate the first 25 eigengenes for each module. Based on these results, our study focused on the above seven modules, specifically analyzing genes with a module membership (kME) greater than 0.3 in each module, yielding a total of 471 genes for further investigation (**Supplementary Table S5**). Ultimately, the intersection of these genes with the DEGs identified 313 candidate genes that participated in FAM upregulation in ccRCC (**Figure 4M**, **Supplementary Table S6**).

### Outcomes of GO and DO enrichment analysis

We used GO and DO analysis to uncover the connections between the 313 FAM genes that were favorably regulated and their functions in different biological systems. The FAM process significantly enhanced candidate genes, according to GO enrichment analysis (**Figure 4N**, **Supplementary Table S7**). The results of the DO enrichment analysis showed that cancer, and ccRCC in particular, had a significant enrichment of candidate genes. (**Figure 4O**).

### Screening of the OFGs by several machine learning algorithms

**Figure 5:**
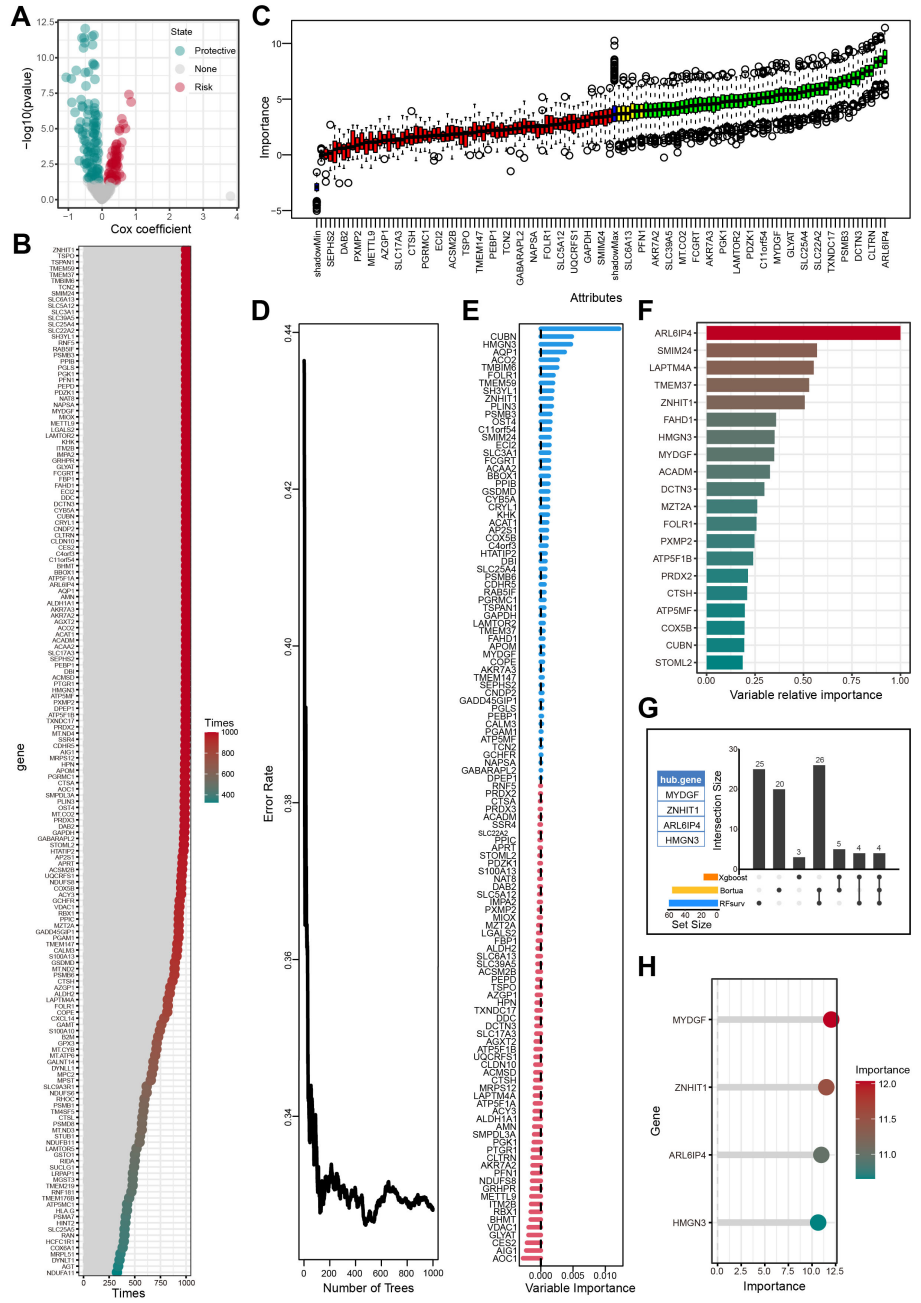
Screening of OFGs by multiple machine learning algorithms. **(A)** Outcomes of univariate Cox regression analysis. **(B)** Outcomes of bootstrap approach. **(C)** Outcomes of Boruta algorithm. **(D-E)** Outcomes of RF algorithm. **(F)** Outcomes of XGBoost algorithm. **(G)** The outcomes of the Venn diagram of the aforementioned 3 machine learning algorithms. **(H)** The importance of feature genes in predicting HFS malignant cells evaluated by the RF algorithm.

Univariate Cox regression analysis identified 166 genes from the 313 candidate genes that were significantly associated with OS (**Figure 5A**, **Supplementary Table S8**). The bootstrap method further chose 124 of 166 prognostic genes that exhibited robustness in sample resampling (**Figure 5B**, **Supplementary Table S9**). Three machine learning algorithms were then employed to optimize the selection of OFGs. The Boruta algorithm removed irrelevant features, resulting in 55 key genes (**Figure 5C**, **Supplementary Table S10**). The RF algorithm identified 59 key genes with importance > 0 (**Figures 5D, E**, **Supplementary Table S11**). The XGBoost algorithm evaluated feature importance and selected 16 key genes with importance > 0.2 (**Figure 5F**, **Supplementary Table S12**). Cross-analysis of the genes selected by all three algorithms revealed four OFGs: MYDGF, ZNHIT1, HMGN3, and ARL6IP4 (**Figure 5G**, **Supplementary Table S13**). Additionally, we innovatively applied the RF algorithm to assess the significance of the OFGs in predicting HFS malignant cells. In this analytical framework, HFS malignant cells were designated the prediction target, while LFS malignant cells were the control group. The results indicated that MYDGF demonstrated strong predictive value (**Figure 5H**, **Supplementary Table S14**).

### Validation of the OFGs at the single-cell level

**Figure 6:**
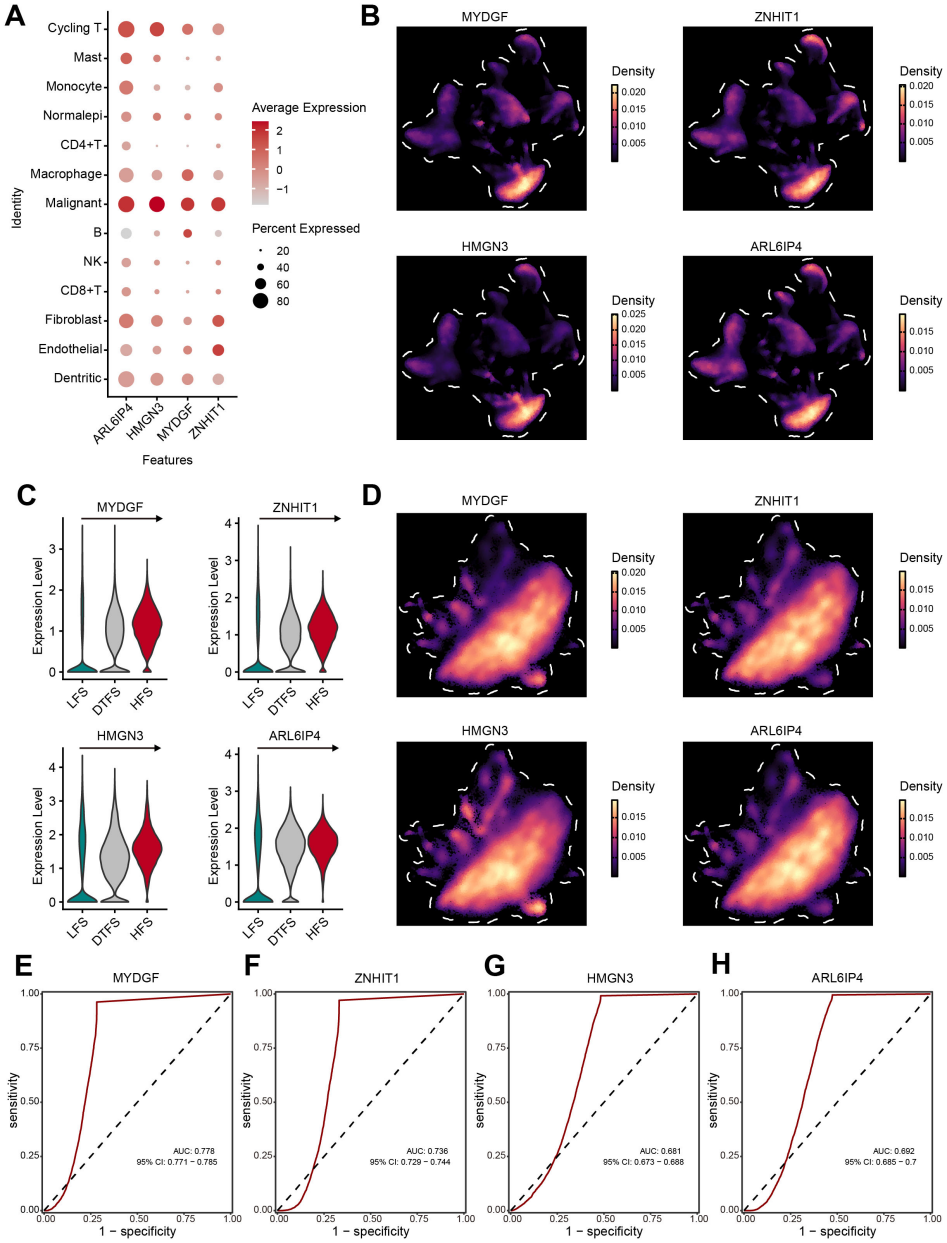
Verification of OFGs at the single-cell level. **(A, B)** The bubble plot and UMAP analysis results demonstrated that all four feature genes had high expression in malignant cells. **(C, D)** Further analysis illustrated that the feature genes had significant expression in HFS malignant cells, showing a gradient increase in expression. **(E–H)** ROC curves estimated the diagnostic performance of feature genes in distinguishing HFS malignant cells from LFS malignant cells.

To further identify the specific cell types affected by the OFGs, we conducted validation at the single-cell level. The overall analysis revealed that the OFGs were most concentrated in malignant cells, with the highest average expression levels (**Figure 6A**). The UMAP plots visually demonstrated that the OFGs showed a predominant expression in malignant cells (**Figure 6B**). In the previously defined HFS, DTFS, and LFS groups, all four genes showed significant enrichment in the HFS group, with an expression showing a clear, dynamic increase (**Figures 6C, D**). The accuracy of the feature genes in predicting HFS was analyzed using ROC curves (**Figures 6E–H**). The results indicated that all four genes exhibited strong diagnostic performance, with MYDGF (AUC = 0.778) demonstrating the highest discriminative ability, followed by ZNHIT1 (AUC = 0.736), HMGN3 (AUC = 0.681) and ARL6IP4 (AUC = 0.692).

### Assessment of prognosis value for OFGs

**Figure 7:**
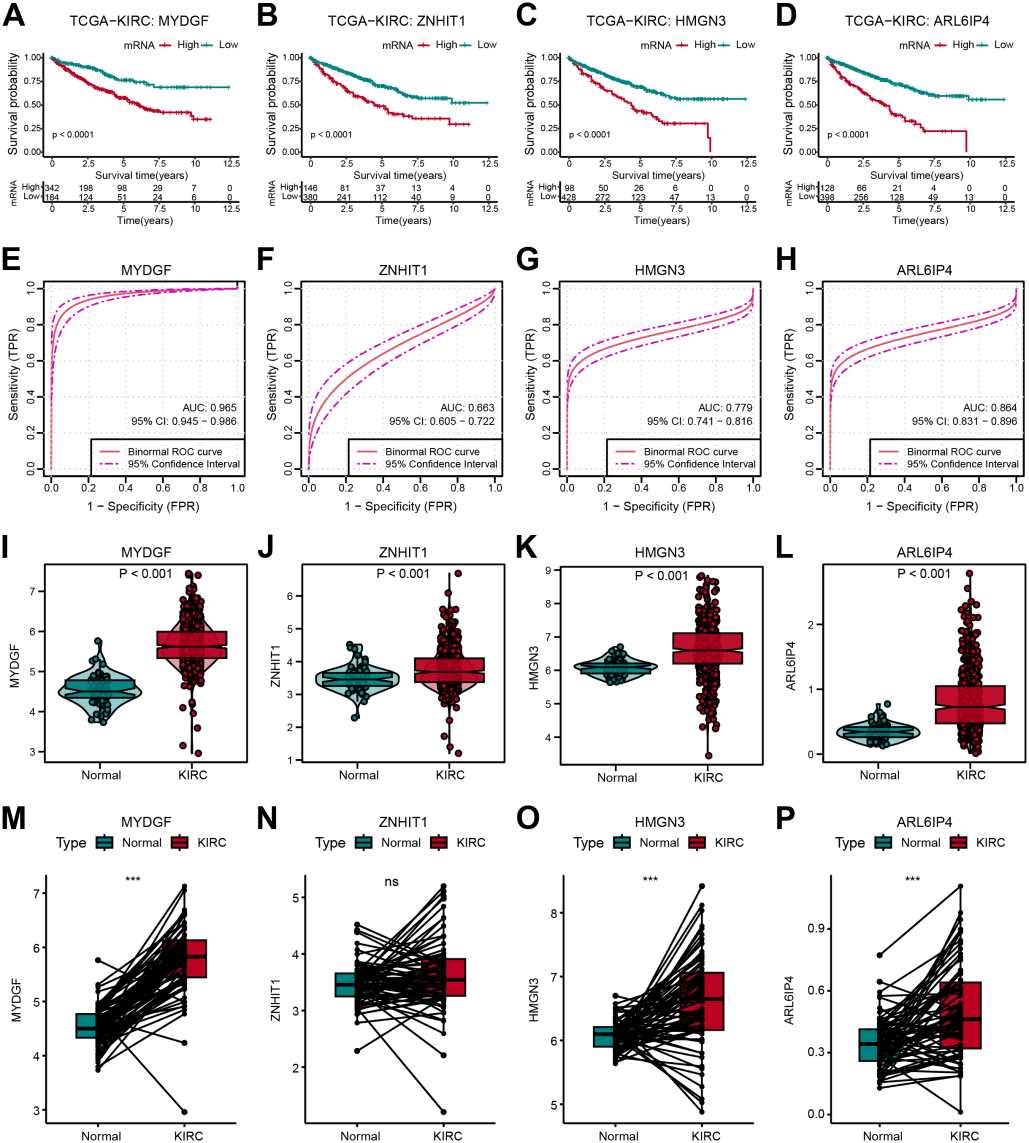
Verification of OFGs in Bulk-Level Analysis. **(A–D)** Kaplan-Meier survival curves for feature genes in TCGA database. **(E–H)** ROC curves for feature genes in TCGA database. **(I–L)** Feature gene expression between tumor and normal samples. **(M–P)** Comparison of feature gene expression between paired tumor and normal samples.

The distribution and predictive efficacy of the OFGs at the bulk level were further clarified. All four genes showed predictive value for OS (**Figures 7A–D**). Their diagnostic value was evaluated through ROC curves, with AUC values of 0.965 for MYDGF, 0.663 for ZNHIT1, 0.779 for HMGN3, and 0.864 for ARL6IP4 (**Figures 7E–H**). Compared to healthy controls from TCGA data, these gene expressions were significantly higher in ccRCC patients (**Figures 7I–L**). Additionally, in paired tissue samples, we observed that except for ZNHIT1, the other three genes were highly expressed in tumor tissues (**Figures 7M–P**).

### Cellular communication and trajectory of MYDGF+ malignant cells

**Figure 8:**
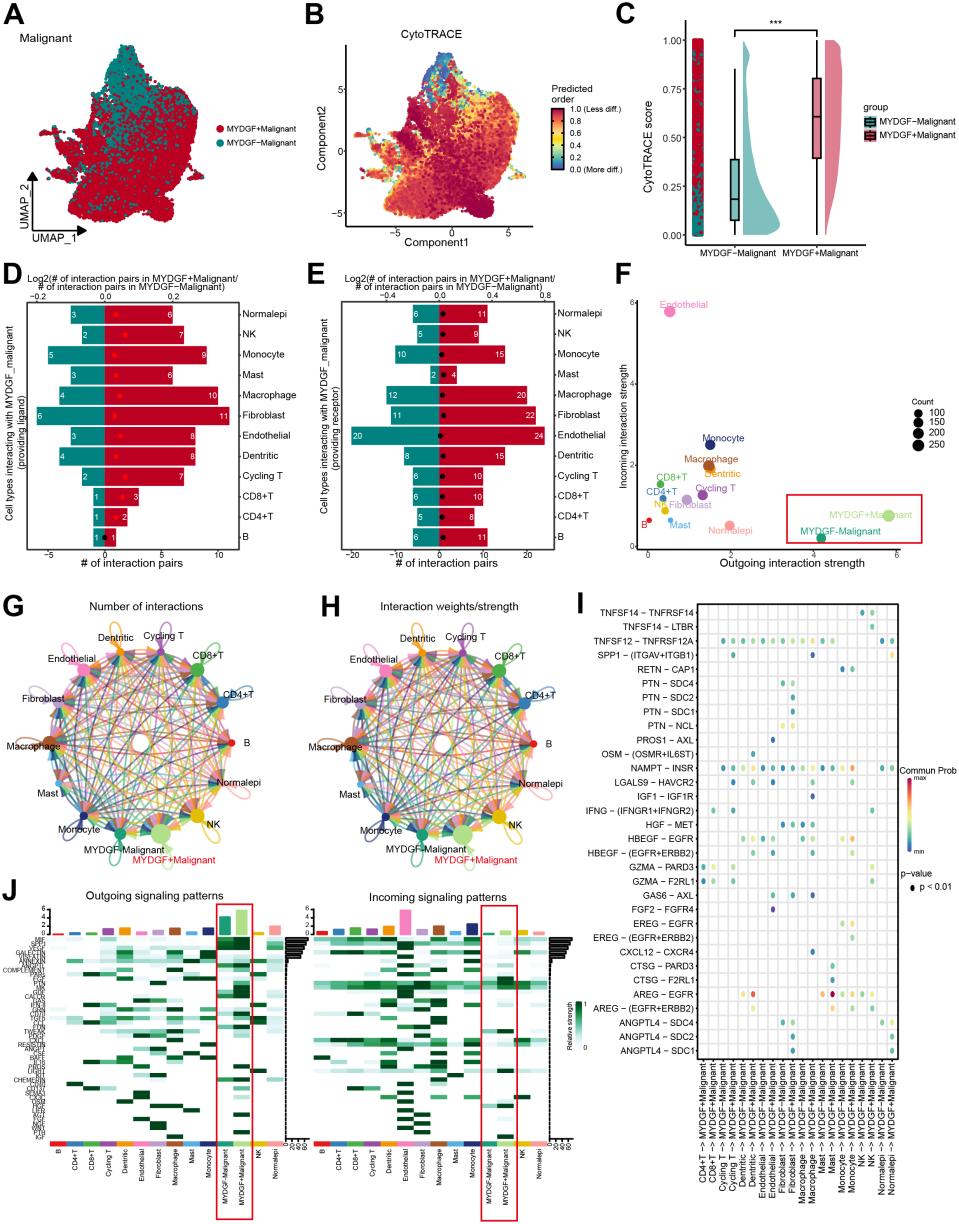
Trajectory analysis and cellular communication in MYDGF+ malignant cells. **(A)** The distribution of MYDGF in malignant cells was visualized by a UMAP plot. **(B)** CytoTRACE analysis of MYDGF+ malignant cells. **(C)** Raincloud plot of CytoTRACE scores in MYDGF+ malignant cells and MYDGF- malignant cells. **(D, E)** Bar plots showed the number of interactions between MYDGF+ malignant cells and other cell types. **(F)** The correlation between differential outgoing contacts and the degree of incoming interactions in MYDGF+ malignant cells and MYDGF- malignant cells. **(G–H)** Quantity and intensity of cellular communications between MYDGF+ malignant cells and other cell types. **(I)** MYDGF+ malignant cells interacting with various cell ligand-receptor bubble diagrams. **(J)** A heat map summarizing the outgoing and incoming signal pathways of MYDGF+ malignant cells and other cell types.

To examine the MYDGF biological role, we classified malignant cells from ccRCC samples into two categories according to MYDGF expression levels: MYDGF+ and MYDGF- cells. The UMAP plot illustrated the distribution of these two cell groups (**Figure 8A**). CytoTRACE analysis revealed a significant increase in tumor stemness features in the MYDGF+ group compared to the MYDGF- group (**Figures 8B, C**). A comprehensive study of the interactions between MYDGF+/MYDGF- cells and other cell types was conducted. The outcomes indicated that compared to MYDGF- cells, the MYDGF+ cells exhibited a significantly higher number of potential ligand-receptor pairs with other cells (**Figures 8D, E**). Cell communication analysis showed that compared to MYDGF- cells, MYDGF+ cells displayed a higher overall level of intercellular communication (**Figure 8F**). **Figures 8G, H** present the count and strength of cell communication between the two groups and other cell types. **Figure 8I** further explores the ligand-receptor interactions between various cell types and MYDGF+/MYDGF- cells. Mast cells showed enhanced communication with MYDGF+ cells through the AREG-EGFR ligand-receptor relationship regarding signal reception. Additionally, heatmap results showed that MYDGF+ cells revealed a greater probability of cell communication. The PTH, AGT, and PROS pathways were more active in MYDGF+ cells than in MYDGF- cells. Among the incoming signals, IGF was predominantly expressed in MYDGF+ cells (**Figure 8J**).

### MYDGF expression was upregulated in human ccRCC tissues and cells

**Figure 9:**
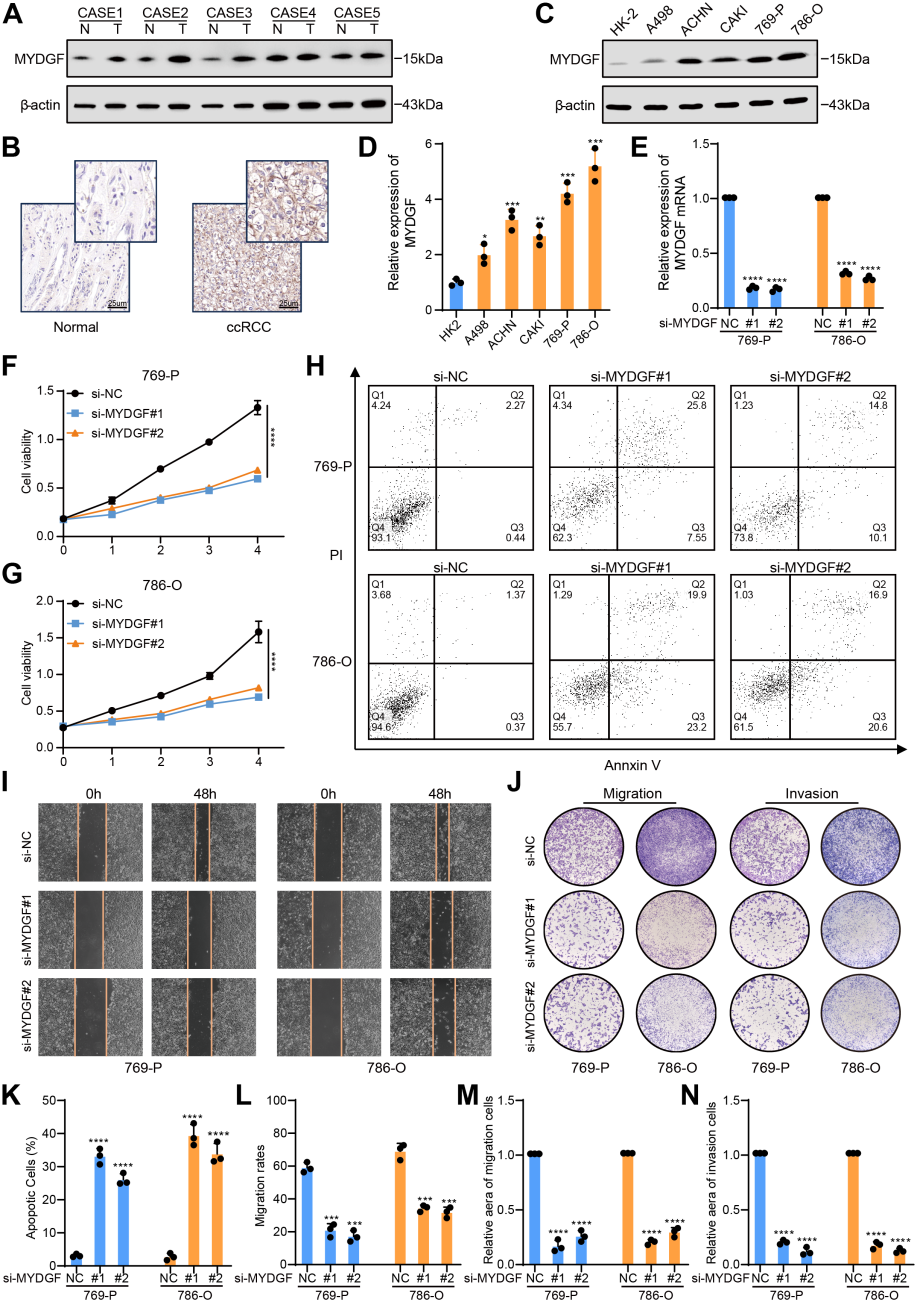
MYDGF promoted cell proliferation, inhibited apoptosis, and suppressed FAO in ccRCC. **(A)** The protein levels of MYDGF were measured in ccRCC and neighboring normal tissues. **(B)** MYDGF protein level in neighboring normal and ccRCC tissues as detected by immunohistochemistry (Scale bar: 25 μm). **(C, D)** The differential expression of MYDGF in HK-2 cell and ccRCC cells validated by western blot. **(E)** Determination of MYDGF knockdown efficiency in 786-O and 769-P cells by qRT- PCR**. (F, G)** CCK-8 assessments were performed to estimate the impact of MYDGF suppression on cell proliferative capacity. **(H)** The apoptosis level was assessed by flow cytometric analysis. **(I)** Representative images of the wound healing assay with MYDGF knockdown. **(J)** Representative images of transwell migration and invasion assays with MYDGF knockdown. **(K)** Statistical results of apopotic cells. **(L)** Statistical results from the wound healing assay. **(M)** Statistical results of the cell migration assay. **(N)** Statistical results of the cell invasion assay. ^*^P < 0.05, ^**^P < 0.01, ^***^P < 0.001 and ^****^P < 0.0001 compared with the control group.

To confirm our previous analysis, we evaluated MYDGF expression in cancerous and adjacent non-cancerous tissues from patients diagnosed with ccRCC. WB analysis illustrated a marked elevation in MYDGF protein levels in the cancerous tissues relative to the normal tissues (**Figure 9A**). In addition, immunohistochemistry staining corroborated these findings by showing elevated MYDGF expression, specifically in ccRCC tissues (**Figure 9B**). Taken together, these outcomes showed that MYDGF was overexpressed in ccRCC tissues relative to adjacent normal tissues. Examining the levels of MYDGF in both normal renal tubular cells (HK-2) and five different ccRCC cell lines (A498, ACHN, CAKI, 769-P, and 786-O) allowed us to explore the MYDGF expression in ccRCC further. Consistent with the tissue-based findings, MYDGF expression was notably elevated in the ccRCC cell lines compared to the normal renal cells (**Figures 9C, D**).

### MYDGF functions as an oncogene in ccRCC

MYDGF protein level showed a significant elevation in the 769-P and 786-O cell lines. Consequently, MYDGF was knocked down in both cell lines, and the effectiveness of the suppression was validated by qRT-PCR (**Figure 9E**). CCK-8 assays revealed that MYDGF knockdown inhibited cell proliferation (**Figures 9F, G**). Flow cytometry analysis indicated that MYDGF knockdown significantly increased apoptosis in ccRCC cells (**Figures 9H, K**). Cell motility was assessed through wound healing (**Figures 9I, L**) and transwell assays (**Figures 9J, M, N**), both of which demonstrated that MYDGF knockdown reduced the migratory capacity of ccRCC cells. In summary, these results suggest that MYDGF promotes ccRCC progression by triggering cell growth and migration while inhibiting cell death.

### MYDGF promotes ccRCC by inhibiting FAO

**Figure 10:**
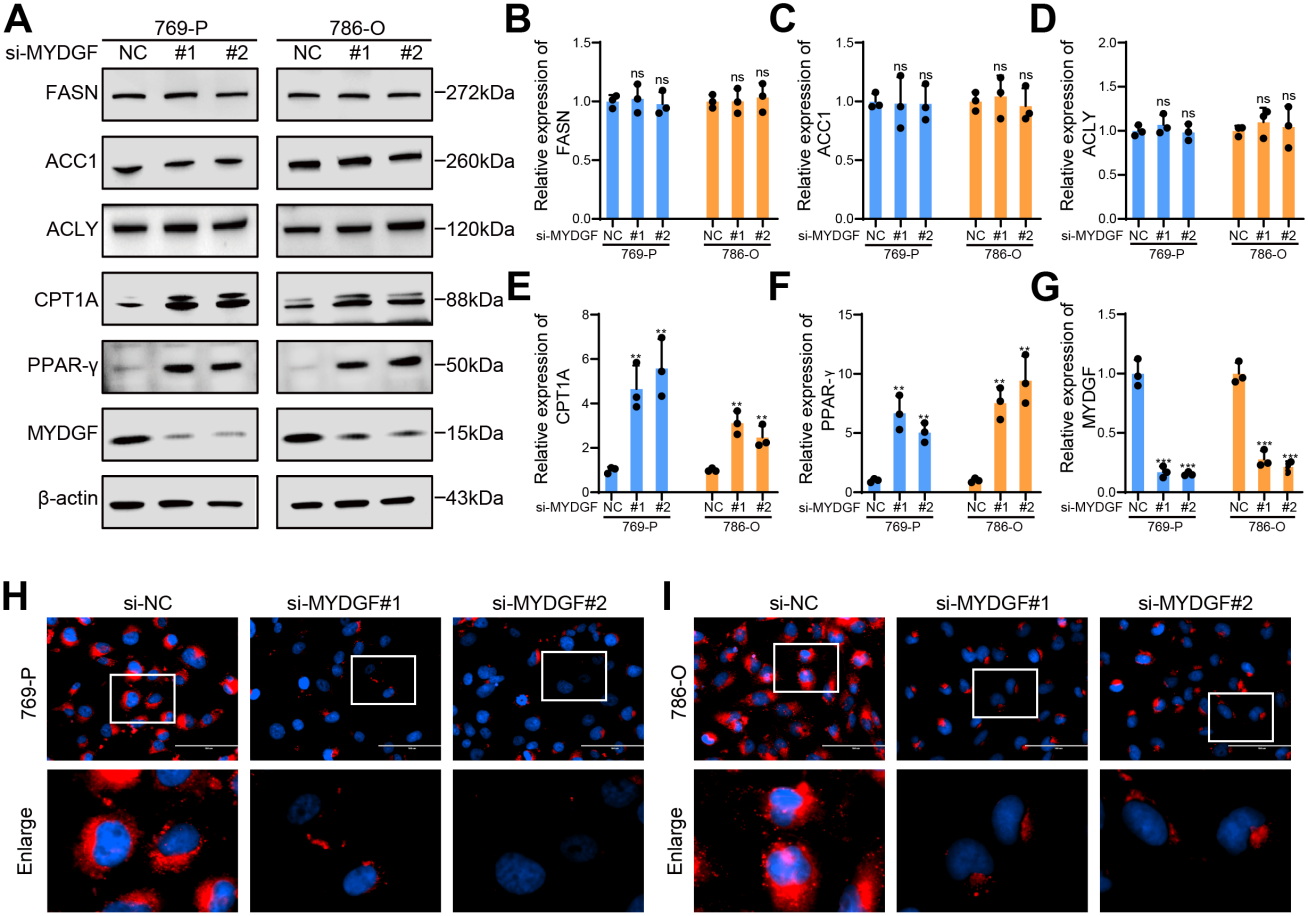
MYDGF suppressed FAO in ccRCC. **(A)** The protein levels linked to FAM were determined by western blot. **(B)** Relative quantitative level of FASN protein. **(C)** Relative quantitative level of ACC1 protein. **(D)** Relative quantitative level of ACLY protein. **(E)** Relative quantitative level of CPT1A protein. **(F)** Relative quantitative level of PPAR-γ protein. **(G)** Relative quantitative level of MYDGF protein. **(H)** Nile Red staining of 769-P cells following MYDGF knockdown (Scale bar: 100 μm). **(I)** Nile Red staining of 786-O cells following MYDGF knockdown (Scale bar: 100 μm). ns (no significance, *P* > 0.05), ^**^P < 0.01 and ^***^P < 0.001 compared with the control group.

To further investigate the role of MYDGF in FAM, we used WB to assess changes in proteins associated with fatty acid synthesis and FAO following MYDGF knockdown (**Figure 10A**). The results demonstrated a significant upregulation of key genes involved in FAO, including PPAR-γ and CPT1A, in the MYDGF knockdown group. Conversely, the levels of major genes that participated in fatty acid synthesis, such as FASN, ACC1, and ACLY, remained unchanged (**Figures 10B–G**). Additionally, Nile Red staining results demonstrated a substantial reduction in intracellular lipid droplets following MYDGF knockdown (**Figures 10H, I**). These findings suggested that MYDGF may facilitate ccRCC progression by inhibiting FAO.

## Discussion

Metabolic reprogramming constitutes a defining characteristic of malignancy, describing the adaptive alterations in cancer cell metabolism that support growth under abnormal conditions (38). In ccRCC, this process is marked by significant disruptions in lipid metabolism (39). Along with the well-characterized disturbances in glucose (Warburg effect) and amino acid metabolism, particularly glutamine, there is increasing recognition of FAM function in tumor development. Dysregulation of FAM has been noted in several malignancies, including kidney, breast, prostate, and lung cancer (40–43). However, the regulatory mechanisms of the FAM pathway in ccRCC remain inadequately explored.

Several investigations have concentrated on FAM function in the diagnosis and prognosis of ccRCC (44, 45). FAM-related gene signatures have been associated with poor OS and resistance to immunotherapy in ccRCC (46). Zhang et al. identified and validated 10 FAM-linked genes for the prediction of ccRCC prognosis (47). Screening FAM-related genes using bulk-RNA-seq was the basis of each of the above investigations. There is cellular heterogeneity inside tumors that whole tissues cannot capture because they reflect average gene expression levels. These constraints have been efficiently addressed by single-cell transcriptomics and ST. Furthermore, There is insufficient elucidation of the function of FAM levels in malignant cells, validation from clinical samples, and investigation into particular processes in these investigations.

Herein, we initially utilized scRNA-seq profiles to evaluate the FAM heterogeneity in ccRCC. Using the obtained FAM gene set combined with five scoring methods, we observed a significant increase in FAM activity in epithelial cells, especially in malignant epithelial cells. ST provided additional evidence supporting FAM heterogeneity. Notably, we detected considerable variability in FAM activity scores within malignant cells, suggesting that FAM heterogeneity exists not only between different cell types but also within malignant epithelial cells. Subsequently, malignant cells were categorized into three groups: LFS, HFS, and DTFS. Functional analysis revealed that the HFS group exhibited enhanced cell communication and greater stemness. Enrichment analysis showed that HFS was associated with more metabolic and cancer-related signaling pathways, highlighting its potential function in promoting tumor development. By integrating key module genes from hdWGCNA and DEGs between the HFS and LFS groups, we identified a candidate gene set linked to FAM upregulation in ccRCC. We applied various machine-learning algorithms to filter the candidate gene set to identify biomarkers related to hierarchical composition and clinical outcomes. Our study identified four OFGs that were correlated with elevated FAM activity. MYDGF was ultimately selected as the primary biomarker for further investigation due to its association with poor prognosis, high tumor expression, and superior predictive capability.

Encoding MYDGF is conducted by the open reading frame 10 on chromosome 19, also called C19orf10 (48). It is named for its secretion by bone marrow-derived monocytes and macrophages, playing a significant function in numerous metabolic disorders and malignancies (49). Studies have shown that MYDGF knockout diabetic mice exhibit raised concentrations of total cholesterol, triglycerides, and free fatty acids, indicating that the absence of MYDGF leads to lipid metabolism disorders. Further research indicates that MYDGF can regulate GLP-1 production and release, improving lipid metabolism in diabetic mice (50). MYDGF has also been linked to the recurrence risk of non-alcoholic fatty liver disease (NAFLD), where its absence worsens liver index, lipogenesis, and liver dysfunction while restoring it mitigates these effects. These findings suggest that MYDGF can suppress inflammation and reduce hepatic lipid synthesis, offering protection against NAFLD (51). MYDGF may promote tumor angiogenesis and macrophage infiltration in hepatocellular carcinoma, releasing inflammatory cytokines, including IL-6 and TNF-α, which accelerate tumor progression (52). Our findings and existing literature underscore the importance of studying FAM regulatory mechanisms in ccRCC and highlight the potential of MYDGF as a therapeutic target. However, the relationship between MYDGF and FAM in ccRCC remains unexplored, warranting further investigation to identify potential pathways.

We investigated the expression of MYDGF in both clinical samples and cell lines of ccRCC. Our findings demonstrated that MYDGF was significantly upregulated in ccRCC, which corroborated our analysis and was consistent with previous reports (53). In MYDGF knockdown ccRCC cells, we observed a marked increase in apoptosis, along with reduced cell proliferation, migration and invasion, indicating that MYDGF contributes to the malignant progression of ccRCC. Additionally, the reprogramming of FAM in ccRCC, including disruptions in fatty acid synthesis and FAO, is pivotal in lipid storage. To further examine MYDGF’s role in FAM, we estimated the key protein levels that participated in this process. The outcomes showed that inhibiting MYDGF substantially elevated the CPT1A and PPAR-γ expression while having no effect on enzymes associated with fatty acid synthesis. These outcomes indicate that MYDGF enhances lipid accumulation by inhibiting FAO, thereby facilitating ccRCC progression.

Despite these promising findings, several limitations must be acknowledged in our research. First, both scRNA-seq and ST methods possess inherent dropout rates, potentially leading to the exclusion of genes with low expression throughout the screening of FAM-related genes. The outcomes on the diagnostic and prognostic value were brought from TCGA, which necessitates large-scale prospective clinical trials for validation. Furthermore, additional *in vivo* and *in vitro* trials are required to explore the biological function and mechanisms of MYDGF.

## Conclusion

In brief, our investigation provided the first comprehensive analysis of FAM heterogeneity and redefined the gene set linked to increased FAM in individuals with ccRCC at the single-cell level. We identified important feature genes associated with FAM by combining this gene set with large-scale RNA-seq data and applying machine learning algorithms. Subsequent experiments validated that MYDGF served as a critical biomarker for FAM, promoting lipid deposition through inhibiting FAO, thereby accelerating tumor progression. These findings offer a promising foundation for personalized treatment strategies to improve outcomes in ccRCC patients.

## Data Availability

The original contributions presented in the study are included in the article/**Supplementary Material**. Further inquiries can be directed to the corresponding authors.
