# Supplementary material for: Cellular hierarchy framework based on single-cell and bulk RNA sequencing reveals fatty acid metabolic biomarker MYDGF as a therapeutic target for ccRCC
Source: Front Immunol. 2025 Jun 5;16:1615601. doi: 10.3389/fimmu.2025.1615601 (PMC12176760; doi:10.3389/fimmu.2025.1615601)
Supplement: Supplementary file 1 [file Table1.docx]

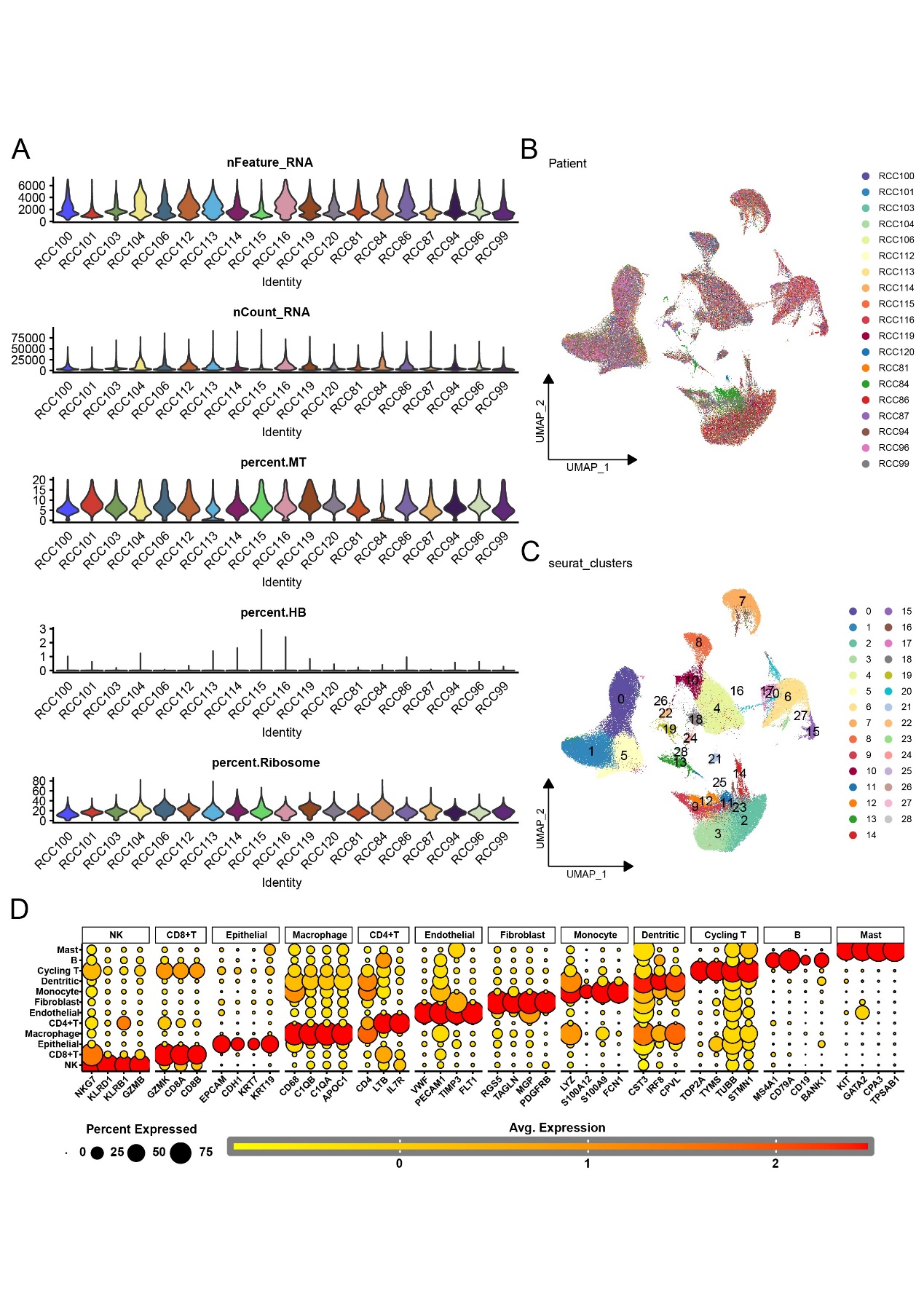


**Figure S1**

Filtering and processing of scRNA-seq data. (A) Quality control for inclusive data. (B) The cell distribution of the samples showed no significant batch effect. (C) The results of UMAP plot indicated that all cells were finely classified into 29 clusters. (D) Representative marker genes for each cell type.


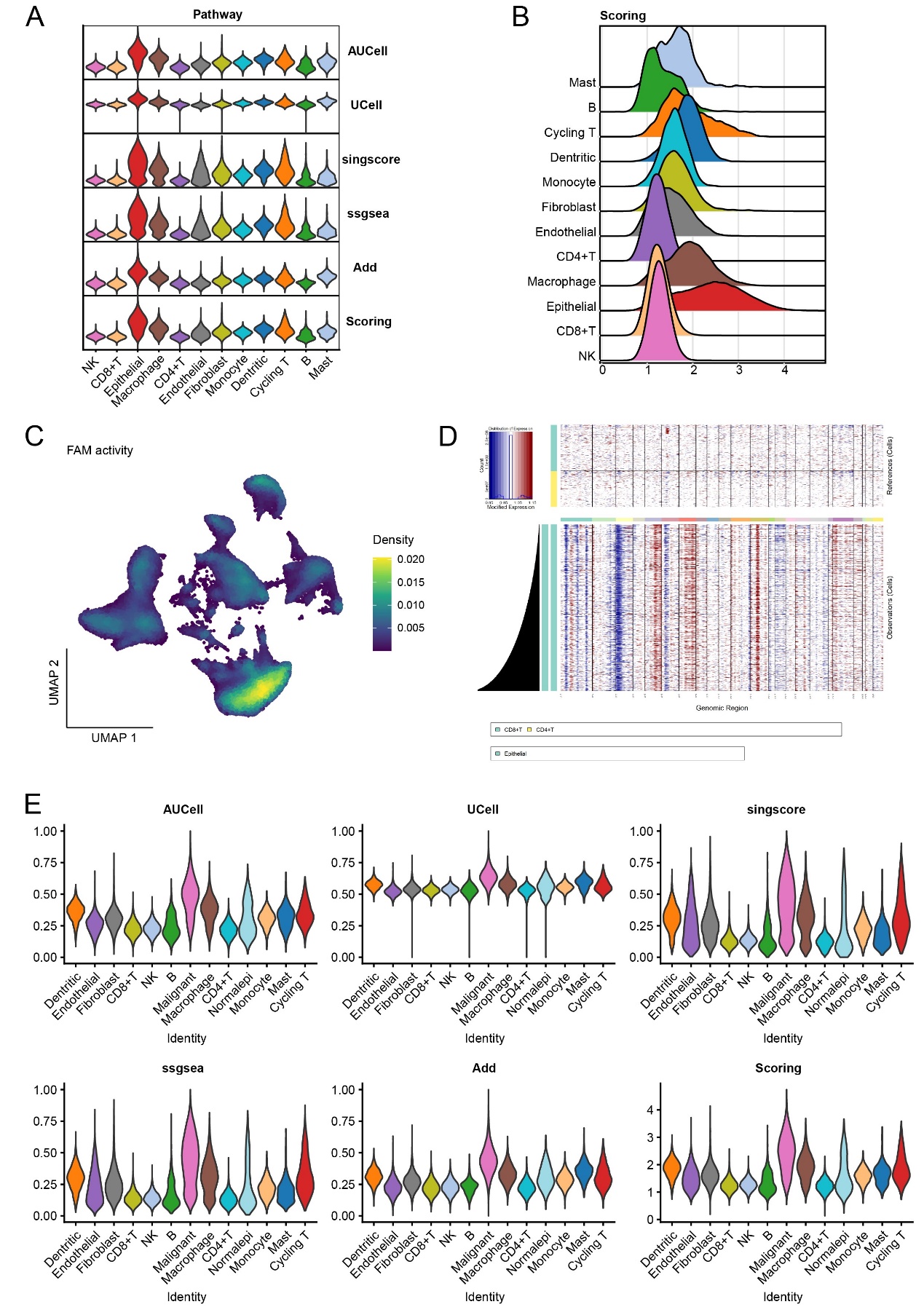


**Figure S2**

Heterogeneity among the expression of FAM gene set. (A-B) Violin plot and density map showed expression scores of FAM activity for each cell type using AUCell, UCell, singscore, ssGSEA and Add algorithms. (C) UMAP plot showed the activity of FAM. (D) Graded heatmap showing CNVs of epithelial cells from tissues of each origin. (E) The results of AUCell, Ucell, singscore, ssGSEA and AddModuleScore algorithms showed that malignant cells had the highest aggregation activity.


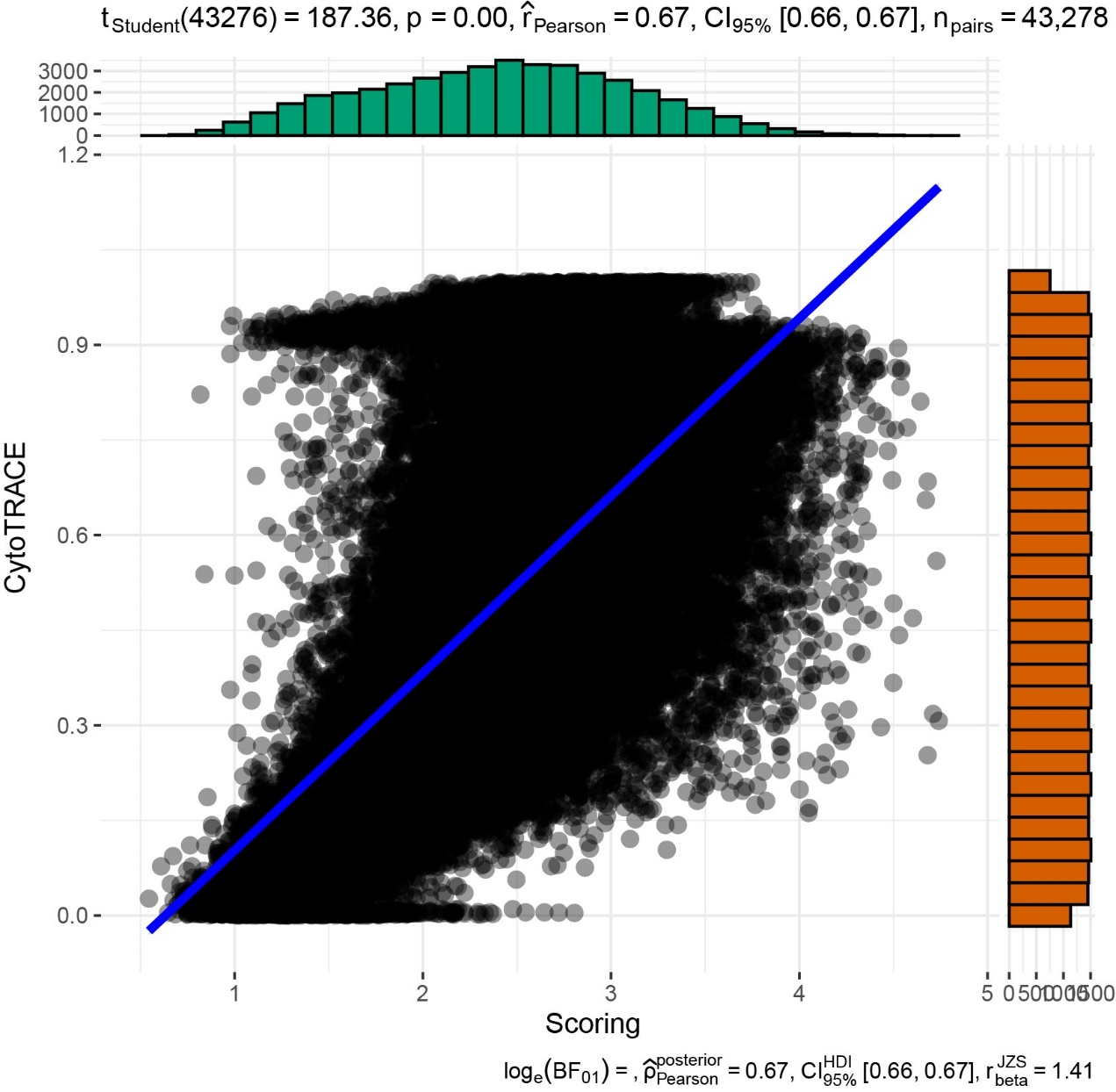


**Figure S3**

Scatter plot of the correlation between CytoTRACE score and FAM score.
